# Accuracy and Outcomes of a Novel Cut-Block Positioning Robotic-Arm Assisted System for Total Knee Arthroplasty: A Systematic Review and Meta-Analysis

**DOI:** 10.1016/j.artd.2024.101451

**Published:** 2024-08-04

**Authors:** Faseeh Zaidi, Craig M. Goplen, Scott M. Bolam, Andrew P. Monk

**Affiliations:** a Department of Surgery, University of Auckland, Auckland, New Zealand; b Auckland Bioengineering Institute, University of Auckland, Auckland, New Zealand; c Department of Surgery, University of Alberta, Edmonton, Alberta, Canada; d Department of Orthopaedic Surgery, Auckland City Hospital, Auckland, New Zealand

**Keywords:** Total knee arthroplasty, Robotic surgery, Accuracy, Systematic review, ROSA

## Abstract

**Background:**

The primary objective of this study was to determine the accuracy and precision of component positioning of the ROSA Robotic System for total knee arthroplasty (TKA).

**Methods:**

A Preferred Reporting Items for Systematic Reviews and Meta-Analysis systematic review was conducted using 4 electronic databases (MEDLINE, EMBASE, Pubmed, and Cochrane Library) to identify all clinical and radiological studies reporting information about the use and results of the ROSA system. The criteria for inclusion were published research articles evaluating the accuracy of component positioning, learning curve, component alignment, complications, and functional outcomes in adults who underwent robotic-assisted TKA. The National Institutes of Health Quality Assessment Tool was used to evaluate the quality of all the included studies.

**Results:**

A total of 26 studies were assessed for eligibility, and 17 met the inclusion criteria. Nine studies reported on the accuracy and precision of component positioning. The ROSA platform for TKA had a cutting error of less than 0.6^°^ for all coronal and sagittal parameters. Pooled analysis demonstrated accuracy within 0.61-1.87^°^ and precision within 0.97-1.34^°^ when the final intraoperative plan was compared to postoperative radiographs with fewer outliers. Four studies reported improved functional scores with ROSA-assisted TKA than conventional TKA within 1 year of surgery. There was no difference in overall complication rates when compared to conventional TKA.

**Conclusions:**

The ROSA system is both highly accurate and precise, with fewer outliers when analyzed at various time points, including postoperative standing radiographs. Future studies with robust methodology and longer follow-up are required to demonstrate whether these findings have any clinical benefits in the long term.

## Introduction

Robotic-assisted total knee arthroplasty (RA-TKA) has gained significant interest in recent years to improve implant placement accuracy and clinical outcomes [[1], [2], [3]]. Numerous platforms are now available with various features and workflows. These platforms can be classified based on the role the surgeon has in directing and the feedback provided by the robot. Fully active robotic platforms perform a task independent of the surgeon within a defined field, providing haptic feedback but often relying on preoperative cross-sectional imaging [4]. Meanwhile, semi-active systems offer a collaborative environment where the surgeon can direct the robotic platform with varying degrees of haptic feedback. These platforms can have different end-effectors, which refers to the device at the robot's end that interacts with the environment. Common end effectors for a RA-TKA platform are a saw blade, burr, or cut block [5].

Recently, a collaborative semi-active platform (ROSA Robotic System, Zimmer Biomet, Montreal, Quebec, Canada) has been available since 2019 that enables a universal cut block to be placed using an advanced robotic arm without requiring preoperative imaging [6]. First, specific anatomical landmarks define and orient the patient’s knee with a platform. Soft tissue assessment is then completed to allow for personalized alignment targets. The surgeon can independently complete bony resections using a conventional oscillating saw through a traditional cut block, which is placed by a robotic arm. The accuracy of the cut is then verified with an intraoperative validation device placed on the cut surface before the implantation of the total knee arthroplasty (TKA) components. The system may also be used with preoperative full-length anteroposterior and lateral radiographs converted to 3-dimensional imaging using X-atlas Platform (Zimmer Biomet, Montreal, Quebec, Canada) to assist with preoperative patient-specific implant positioning [7].

Studies have demonstrated that this platform can be accurate and precise [6,[8], [9], [10], [11], [12]]. Accuracy is defined as the ability to achieve a planned target, whereas precision is defined as the ability to reproduce the same result repeatedly. However, studies using this platform have used various reference points to define the planned target. While some studies reference the accuracy of the bony resection in relation to the anatomical reference points, few studies have defined the accuracy and precision of implant placement based on postoperative radiographs compared to the intraoperative planned position. In addition, this collaborative semi-active platform's impact on clinical outcomes remains unclear, as studies have used various alignment targets, including mechanical, functional, and kinematic alignment methods for comparison.

This systematic review and meta-analysis were conducted to define the accuracy and precision of component positioning on this collaborative semi-active platform that places conventional cut blocks. The secondary objectives are to determine the clinical outcomes, accounting for the alignment strategy, associated learning curve, and complications associated with the ROSA Robotic System for TKA.

## Material and methods

This systematic review and meta-analysis was conducted in accordance with the Preferred Reporting Items for Systematic Reviews and Meta-Analysis guidelines [13]. This study was registered with the International Prospective Register of Systematic Reviews (ID no. CRD42023438619) prior to database searching and study retrieval.

### Search strategy

A comprehensive literature search was conducted in June 2023 across 4 electronic databases: 1) Ovid MEDLINE(R) Epub Ahead of Print, In-Process & Other Non-Indexed Citations, Ovid MEDLINE(R) Daily and Ovid MEDLINE(R); 2) EMBASE; 3) PubMed; and 4) Cochrane Library, without date restriction. The search strategy included but was not limited to the following terms: “Arthroplasty, Replacement, Knee,” “Total Knee Arthroplasty,” “Total Knee Replacement,” “Robotics,” “Robot-assisted,” and “Robotic Surgical Procedure.” Restrictions were applied to the search to only include studies in the English language. A gray literature search (Google and Google Scholar) was conducted to identify nonindexed studies not appearing in the above databases. The complete search strategy employed for all 4 databases is demonstrated in Appendix A.

### Study selection and inclusion criteria

The inclusion criteria were studies evaluating the accuracy of implant positioning, operating time, prosthesis alignment, complications, and functional outcomes in adults who underwent RA-TKA with the ROSA Robotic System. Any studies that did not include RA-TKA with ROSA or investigated other surgical techniques, such as unicompartmental knee arthroplasty, were excluded. Retrospective, prospective, and cadaveric studies were incorporated into the review process. Systematic reviews, case reports, methodological or technique studies, ongoing randomized controlled trials (RCTs), conference abstracts, comments, newspaper articles, editorials, and letters were excluded. Studies were also excluded if they were not available in the English language.

### Data extraction

One investigator (F.Z.) imported all retrieved studies into Covidence, a web-based collaboration software for data extraction from systematic and other literature reviews [14]. All identified article titles and abstracts were screened for eligibility independently by 2 authors (F.Z. and C.G.). Duplicate articles were excluded. The reference lists of all relevant articles were reviewed to identify additional relevant studies. The full texts of all potentially relevant articles were analyzed to further assess their eligibility. Data extracted included study design, publication date, country of publication, sample size, control of confounding, follow-up, type of implant used, preoperative surgical data including radiograph protocols, and imageless or image-based planning. Intraoperative and postoperative data extracted included measures of accuracy and precision, complications, and clinical outcome measures. Each reviewer then cross-checked all data, and disagreements between reviewers were discussed and resolved by consensus; no third party was required to achieve consensus. Authors were contacted if the data was incomplete or missing.

### Outcome measures

The primary objectives were to report the coronal and sagittal TKA implant accuracy and precision of positioning when utilizing ROSA. Accuracy relates to how close the measurement is to the predefined target and was measured by the mean difference between 2 measurements. Precision represents variability and was measured by the standard deviation. Coronal and sagittal alignment parameters were standardized to the lateral distal femoral angle, medial proximal tibial angle, femoral flexion angle, and tibial slope.

Our secondary objectives were to determine functional outcomes with patient-reported outcome measures (PROMs) and range of motion (ROM) after ROSA-assisted TKA, the learning curve, and specific ROSA-associated TKA complications. Specific complications are described as those directly related to the use of the ROSA Robotic System such as wound complications associated with pin sites used for intraoperative planning and reference, periprosthetic fractures, or fractures at pin sites.

### Quality assessment

Using the National Institutes of Health Quality Assessment Tool for Observational and Cross-Sectional Studies [15], all included publications were reviewed independently for potential risk of bias by 2 authors (F.Z. and C.G.). The assessment tool uses 14 questions to allocate a score to each article (poor, fair, or good). If there was disagreement regarding the scoring of a study, consensus was met after discussion between both reviewers.

### Statistical analysis

Accuracy was all reported as mean difference between the final planned target and recorded data point, while standard deviation of this measurement represented precision as previously described [16]. Descriptive statistics described inconsistent outcomes across studies and were inappropriate for a meta-analysis. Pooled mean and standard deviation were calculated for outcomes that were comparable at specific timepoints. All statistical operations were performed with PRISM 8 (GraphPad, San Diego, CA).

## Results

### Study characteristics

**Figure 1:**
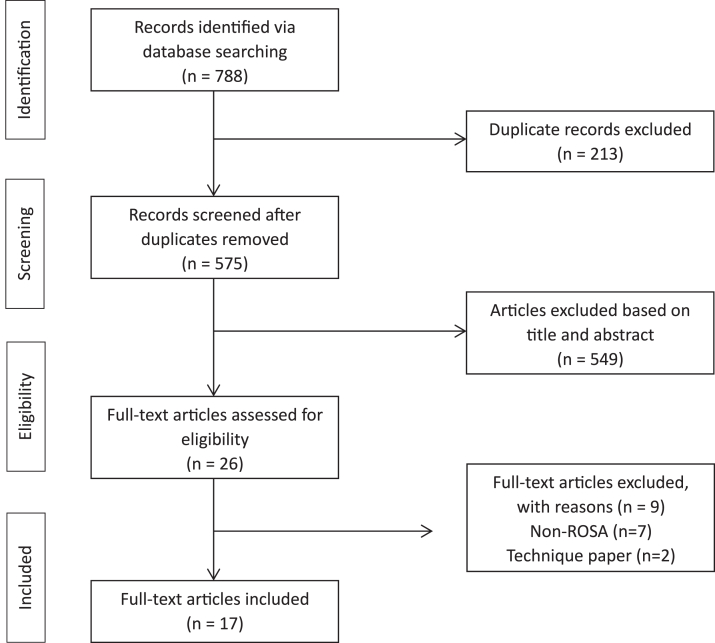
Complete PRISMA flow diagram showing the identification, screening, eligibility, and inclusion process.

The initial search of databases yielded 788 articles. After the initial screening of titles and abstracts, 26 articles met the eligibility criteria for review (Fig. 1). After the final full-text review, a total of 17 articles were included for data extraction. All studies included the ROSA Total Knee System. The year of publication ranged from 2019 to 2023. The reported follow-up periods ranged from a minimum of 2 weeks-21 months. Of the 17 included studies, 3 were prospective, 11 were retrospective, and the remainder were cadaveric studies. Nine studies were controlled with a TKA group performed by conventional manual or navigated techniques. A complete description of the included studies is summarized in Table 1.

**Table 1:** Studies included in the systematic review according to when they were published, design, patient demographics, exclusion criteria, control of confounding, and follow-up.

Authors	Year	Country	Design	Patients (n)	Age (years)	Exclusion criteria	Control of confounding	Follow-up
Batailler et al. [10]	2023	France	Prospective cohort	20 RA-TKA, 20 cTKA	RA-TKA: 60.8 cTKA: 60.7	Previous femoral or tibial osteotomy, preoperative extra-articular deformity >10^°^	Matching (age, BMI, sex)	RA-TKA: 8.4 ± 1.0 mo cTKA: Minimum 6 mo
Bolam et al. [17]	2022	New Zealand	Retrospective cohort	53 RA-TKA, 83 cTKA	70.3	Conversion from UKA to TKA, infection, neurological dysfunction limiting knee mobility, post-traumatic OA with severe knee deformity	-	21.3 mo ± 9 mo
Charette et al. [18]	2022	USA	Cadaveric study	2 specimens	-	No prior surgery on the tested extremity, no appreciable cartilage degeneration, or damage on visual exam	-	-
Haffar et al. [19]	2021	USA	Prospective cohort	20 RA-TKA, 20 cTKA	RA-TKA: 67.8 cTKA: 65.2	Patellar preparation, cementation, closure	Multivariate regression analysis (age, BMI, range of motion)	-
Hasegawa et al. [20]	2022	Japan	Retrospective cohort	38 RA-TKA	73.0	-	-	2 wk
Kenanidis et al. [9]	2023	Greece	Prospective cohort	30 RA-TKA, 30 cTKA	RA-TKA: 69.3 cTKA: 69.1	Complex primary or revision TKA, different knee implants	Matching (age, sex, BMI)	6 mo
Kenanidis et al. [21]	2023	Greece	Retrospective cohort	100 RA-TKA, 100 cTKA	RA-TKA: 74.4 cTKA: 73.6	Revision TKA, different knee implant, patients <18 y	Matching (age, sex, BMI)	-
Khan et al. [22]	2023	USA	Retrospective cohort	254 RA-TKA, 762 cTKA	-	Bilateral simultaneous TKA, revision or conversion TKA	Propensity Matching (age, sex BMI, Charlson comorbidity)	14 mo
Lonner et al. [23]	2022	USA	Retrospective cohort	131 RA-TKA	64.0	Complex primary TKA, revision TKA	Matching (age, sex, BMI)	6 wk
Mancino et al. [6]	2023	Italy	Retrospective Cohort	50 RA-TKA, 47 cTKA	RA-TKA: 69.2 cTKA: 72.4	Varus deformity >15^°^, valgus knee, ligament insufficiency, bone loss requiring augments, neurological disorder, incomplete follow-up	Linear regression (surgical technique, age, sex, BMI)	RA-TKA: 13.4 ± 1.3 months cTKA: 13.7 ± 1.2 mo
Parratte et al. [8]	2023	France	Retrospective case control	40 RA-TKA, 40 cTKA	RA-TKA: 60.0 cTKA: 60.4	Genu valgum >7^°^, extra-articular deformity >10^°^, preoperative patellar maltracking, previous tibial or femoral osteotomy.	Matching (age, sex, BMI)	RA-TKA: 14.1 ± 2.5 months cTKA 15.2 ± 3.3 mo (minimum 12 months)
Parratte et al. [24]	2019	France	Cadaveric study	15 RA-TKA	78.7	-	-	-
Rossi et al. [12]	2023	Italy	Retrospective cohort	75 RA-TKA	70.6	First 10 cases	-	3 mo
Schrednitzki et al. [11]	2023	Germany	Retrospective cohort	71 RA-TKA, 307 cTKA	RA-TKA: 68.4 cTKA: 68.9	-	-	-
Seidenstein et al. [25]	2021	USA	Cadaveric study	14 RA-TKA, 20 cTKA	RA-TKA: 77.0 cTKA: 79.0	-	-	-
Shin et al. [26]	2022	USA	Retrospective cohort	37 RA-TKA	67.4	Patients <45 y, flexion contracture >15^°^, patients with any medical condition or personal circumstances that would prevent completion of follow-up visits	-	-
Vanlommel et al. [27]	2021	Belgium	Retrospective cohort	90 RA-TKA, 90 cTKA	RA-TKA: 68.7 cTKA: 69.8	Patients with congenital deformity, underlying neurological dysfunction, severe deformity (>15^°^ of preoperative varus/valgus alignment or a noncorrectable deformity), prior infection or osteotomy around the knee, prior UKA or osteotomy, or fracture as the primary indication, conversion of UKA to TKA	ANOVA models, which included a group effect (cTKA, learning RA-TKA, master RA-TKA) and surgeon effect	3 mo

RA-TKA, robotic-assisted total knee arthroplasty; cTKA, conventional total knee arthroplasty; UKA, unicompartmental knee arthroplasty; OA, osteoarthritis; BMI, body mass index.

A total of 10 studies reported on the accuracy and precision of ROSA (Table 2). 1 study was image-based [20]. Three studies compared the final implant position measured on full-length standing postoperative radiographs to the final intraoperative plan (Table 3). Seven studies compared the final intraoperative plan to intraoperative validation using the robotic system, with 3 of these not using an external method of validation such as postoperative radiographs.

**Table 2:** List of included studies and outcome measures assessed.

Author	Year	Image-based	Accuracy	Functional outcomes	Learning curve	Complications
Batailler et al. [10]	2023			+		+
Bolam et al. [17]	2022				+	
Charette et al. [18]	2022		+			
Haffar et al. [19]	2021					
Hasegawa et al. [20]	2022	+	+			
Kenanidis et al. [9]	2023			+		+
Kenanidis et al. [21]	2023				+	
Khan et al. [22]	2023			+		
Lonner et al. [23]	2022		+			
Mancino et al. [6]	2023		+	+		
Parratte et al. [8]	2023			+		
Parratte et al. [24]	2019		+			
Rossi et al. [12]	2023		+			
Schrednitzki et al. [11]	2023		+			
Seidenstein et al. [25]	2021		+			
Shin et al. [26]	2022		+			
Vanlommel et al. [27]	2021		+

**Table 3:** Timepoints at which ROSA-assisted TKA accuracy and precision were measured among the included studies.

Author	Year	Planned vs postoperative radiographs	Planned vs intraoperative validation	Intraoperative validation vs postoperative radiographs	Planned vs intraoperative bone measurements
Charette et al. [18]	2022				+
Hasegawa et al. [20]	2022		+		
Lonner et al. [23]	2022		+		
Mancino et al. [6]	2023	+			
Parratte et al. [24]	2019		+	+	+
Rossi et al. [12]	2023	+	+		+
Schrednitzki et al. [11]	2023				+
Seidenstein et al. [25]	2021		+		+
Shin et al. [26]	2022	+	+		
Vanlommel et al. [27]	2021		+		

### Accuracy and precision

The mean difference between the final planned target (whether neutral mechanical alignment or other alignment strategy) and measured data point defined accuracy, while the standard deviation represented the precision in all studies. The pooled accuracy for all coronal and sagittal parameters was less than 1^°^ and precision less than 2^°^ (Table 4). The robotic platform was most accurate and precise when the final intraoperative plan was compared to the validated cut. In contrast, it was less accurate and precise when the final intraoperative plan was compared to the postoperative radiographs.

**Table 4:** Pooled accuracy (mean) and precision (±SD) of ROSA-assisted TKA at different time points.

Parameter	Planned vs postoperative radiographs	Planned vs intraoperative radiographs	Intraoperative validation vs postoperative radiographs
LDFA	0.61 (±0.97)	0.38 (±0.42)	0.60 (±0.10)
MPTA	0.61 (±1.26)	0.52 (±0.58)	0.40 (±1.8)
Tibial slope	0.75 (±1.34)	0.66 (±0.70)	0.10 (±1.7)
Femoral flexion	1.87 (±1.11)	0.52 (±0.60)	0.20 (±0.90)

SD, standard deviation; LDFA, lateral distal femoral angle; MPTA, medial proximal tibial angle.

There were 4 studies [11,12,24,25] that measured the difference between the thickness of intraoperative resection, accounting for the thickness of the saw blade, when compared to the intraoperative plan. Pooled medial and lateral distal femur resection accuracy and precision were 0.53 ± 0.49 mm and 0.41 ± 0.56 mm, respectively. Pooled medial and lateral posterior femur resection accuracy and precision were 0.15 ± 0.52 mm and 0.46 ± 0.44 mm, respectively. Pooled medial and lateral proximal tibia resection accuracy and precision were 0.95 ± 1.09 mm and 0.88 ± 1.19 mm, respectively.

Hasegawa et al. [20] compared cross-sectional 3-dimensional and 2-dimensional imaging after ROSA-assisted TKA. The authors showed that cutting errors were below 0.6^°^, except for the femoral sagittal angle (1.0^°^), and that 3-dimensional measurements were more accurate than 2-dimensional measurements.

Eight studies [8,10,11,20,[24], [25], [26], [27]] reported on the accuracy and precision of the hip-knee-ankle (HKA) angle by determining the percentage of outliers (defined as >3^°^) compared to the planned HKA angle (Table 5). All studies reported decreased rates of outliers when ROSA-assisted TKA was compared to conventional TKA.

**Table 5:** Percentage of outliers (>3^°^ of planned) of the included studies reporting on accuracy and precision of the hip-knee-ankle (HKA) angle after ROSA-assisted TKA.

Author	Year	RA-TKA	Control group	*P* value
Batailler et al. [10]	2023	2/40 (5%)	12/40 (30%)	.003
Hasegawa et al. [20]	2022	0/36 (0%)	-	-
Parratte et al. [8]	2023	4/40 (10%)	8 (20%)	>.05
Parratte et al. [24]	2019	0/30 (0%)	-	-
Schrednitzki et al. [11]	2023	0/71 (0%)	75/308 (24.3%)	<.001
Seidenstein et al. [25]	2021	0/14 (0%)	5/20 (25%)	-
Shin et al. [26]	2022	4/37 (11%)	-	-
Vanlommel et al. [27]	2021	3/58 (5.2%)	19/79 (24.1%)	.003

### Learning curve

There were 3 studies [17,21,27] that reported on the learning curve associated with the implementation of ROSA. Kenanidis et al. [21] conducted a single-surgeon cohort study and reported that 70 cases were required, when compared to a surgeon-matched cohort of conventional TKA, to achieve a similar mean operative time of 68.5 minutes with no increase in complications. Bolam et al. [17] reported an inflexion point using cumulative summation analysis of 8.7 cases (5, 6, and 15 cases individually) when comparing the surgical time between ROSA-assisted TKA and conventional TKA among 3 surgeons. Cumulative summation analyses allow more detailed information to be obtained on incremental changes in outcomes with consecutive cases until predefined levels of surgical proficiency are achieved. Vanlommel et al. [27] reported that among the 3 surgeons with minimal (<20 cases) or no previous RA-TKA experience, the learning curve was 6, 10, and 11 cases for each surgeon.

### Functional outcomes

A total of 5 studies [6,[8], [9], [10],^22^] reported on PROMs after ROSA-assisted TKA (Table 6). Of these, 4 compared outcomes against conventional manual TKA, while one compared ROSA-assisted TKA with navigated TKA. Various alignment targets for both RA-TKA and control groups were used, and only one study was compared against the control group using the same alignment target. No clinically important differences in PROMs were reported within 3 months of surgery. Two studies [8,9] reported improved PROMs at 6 months, and 2 studies [6,8] reported improved PROMs at 1 year when compared to the control group that reached a threshold determined for detecting a clinical difference (Table 6). ROM was assessed in 3 studies [6,8,10], and a significant difference was only reported in one study [6] at 1 year after surgery when ROSA-assisted TKA was compared to navigated TKA.

**Table 6:** Summary of included studies reporting on functional outcomes at specific time points and complications after ROSA-assisted TKA.

Authors	Implant	RA-TKA alignment target	Control group	Control group alignment target	Outcome measure	4-6 weeks	3 months	6 months	1 year	Complications
Batailler et al. [10]	Cemented Persona PS	Functional alignment	Conventional manual TKA	Not reported	KSS Pain	No difference	Not reported	No difference	Not reported	Delayed pin tract healing (n = 1)
					KSS Function	No difference		93.3 vs 80.7 (*P* < .0001)		
					ROM	128.4^°^ vs 120.8^°^ (*P* = .0016)		No difference		
Khan et al. [22]	Persona CR, PS, MC, UC, CPS	Not reported	Conventional manual TKA	Not reported	KOOS-JR	63.1 vs 59.0 (*P* = .35)	Not reported	No difference	77.8 vs 74.3 (*P* = .014)	-
					Delta KOOS-JR	77.8 vs 74.3 (*P* = .014)		No difference	No difference	
Kenanidis et al. [9]	Cemented Nexgen PS	Neutral mechanical alignment	Conventional manual TKA	Neutral mechanical alignment	OKS	Not reported	No difference	37.8 vs 34.8 (*P* = .006)	Not reported	-
					FJS		Not reported	71.6 vs 61.9 (*P* < .001)^a^		
					VAS		No difference	1 vs 2 (*P* = .025)		
Mancino et al. [6]	Persona PS	Functional alignment	Navigated TKA	Neutral mechanical alignment	KSS Pain	Not reported	Not reported	Not reported		-
					KSS Function				119.4^°^ vs 107.1^°^ (*P* < .0001)^a^	
					KOOS Pain				85 vs 79.1 (*P* = .028)	
					KOOS Function				No difference	
					FJS				No difference	
					ROM				No difference	
Parratte et al. [8]	Cemented Persona PS	Functional alignment	Conventional manual TKA	Adjusted mechanical alignment	Delta KSS Knee	Not reported	Not reported	59.3 vs 49.3 (*P* = .003)	No difference	-
					Delta KSS Function			51.7 vs 20.8 (*P* < .001)^a^	48 vs 29.5 (*P* < .004)^a^	
					ROM			No difference	No difference	

PS, posterior stabilized; CR, cruciate retaining; MC, medial congruent; CPS, constrained posterior stabilized; KSS, knee society score; KOOS-JR, knee injury and osteoarthritis outcome score for joint replacement; OKS, Oxford knee score; FJS, forgotten joint score.

a Minimal clinically important difference (MCID)/minimal detectable change (MDC) achieved; delta represents the change in preoperative and postoperative score.

### Complications

No studies reported any difference in complication rates when ROSA-assisted TKA was compared to conventional TKA. 1 study [10] reported a superficial pin tract infection using ROSA registration pin sites outside the surgical wound (Table 6). No studies reported any pin site fractures in the tibia or femur.

### Risk of bias

All included studies were considered “fair” according to the National Institutes of Health Quality Assessment Tool for Observational and Cross-Sectional Studies Checklist for Cohort Studies (Appendix B).

## Discussion

The key finding of this review is that the semi-active collaborative ROSA Knee System exhibited high accuracy and precision. The ROSA robotic platform was most accurate when executing the distal femur coronal cut. In contrast, the sagittal parameters (both femoral flexion and tibial slope) demonstrated the lowest accuracy and precision. When the overall alignment as measured by the HKA was compared to conventional TKA techniques, ROSA-assisted TKA appeared to have much greater accuracy and precision. Conventional TKA had up to 30% of ‘outlier’ cases outside the preferred error range of a ± 3^°^ target window [28], compared to 0%-11% after ROSA-assisted TKA.

Most studies in this systematic review compared the final intraoperative plan to the validated bone resection measured intraoperatively after the bony resection but prior to implant placement (Table 3) [8,12,20,23,[25], [26], [27]]. This comparison resulted in higher accuracy and precision compared to the accuracy and precision of ROSA when the final intraoperative plan was compared to postoperative radiographs. This finding is not unexpected as there are more potential sources of error, which may be additive as more steps are added between comparisons and limit the usefulness of these findings.

Sources of errors when the final intraoperative plan is compared to the validated bone resection include errors intrinsic to the robotic arm, cut block placement, and bone resection through the cut block. It has been reported that bone cutting errors in TKA using slotted cut blocks can range from 0.4-0.8^°^ in the coronal plane and 1.3^°^ in the sagittal plane [29]. In our study, accuracy was greater than 0.8^°^ with certain cuts, suggesting other errors in the system such as the intrinsic robotic arm itself including joint or kinematic errors. However, accuracy and precision of ROSA were comparable to other platforms that do not use traditional cut blocks, such as the semi-active MAKO robotic system (MAKO Robotic Interactive Orthopaedic System, Stryker, USA) [30].

When the final intraoperative plan is compared to postoperative radiographs, additional sources of errors include the registration of the anatomical landmarks. This step is critical to orient the platform to the patient’s knee and the platform's virtual model. While the anatomical registration of ROSA has been reported to be highly accurate and precise in cadavers [18], in vivo registration has never been reported. Other sources of error introduced into this step include cementation of components and coronal and sagittal measurements on postoperative standing radiographs. While standing long-leg radiographs demonstrate a good correlation to computed tomography scans, rotation of a limb can change the distal femur and proximal tibial coronal alignment measurements by 2^°^. This finding was supported by Hasegawa et al. [20], who reported that 3-dimensional imaging was more accurate in measuring final implant placement when compared to plain films among patients who underwent TKA, but the effect size was minimal. Notably, there was variability in the study’s measurement protocol among the included studies. Shin et al. [26] reported unsatisfactory sagittal plane accuracy among patients undergoing RA-TKA. However, the study’s measurement protocol may have been unreliable as it was inconsistent with the manufacturer's recommendations, and the results should be interpreted with caution [31].

ROSA-assisted TKA improved clinical outcomes (PROMs and ROM) compared to conventional TKA among the included studies. Both Parratte et al. [8] and Batailler et al. [10] reported improved Knee Society Scores at 6 months when compared to conventional manual TKA that reached a clinically important threshold. At 1 year, these differences were again noted when the change in the Knee Society Scores function score was analyzed. Mancino et al. [6] also reported that 1-year Knee Injury and Osteoarthritis Outcome Score pain scores and ROM reached a detectable clinical difference when ROSA-assisted TKA was compared to navigated TKA. While Kenanidis et al. [9] did not detect any clinical difference when ROSA-assisted TKA was compared to conventional TKA, they did report a statistical improvement in the Oxford Knee Score and Forgotten Joint Score at 1 year, in addition to more patients willing to undergo the operation again when the 2 groups were compared. However, no differences existed among the included studies when PROMs were compared at 3 months postsurgery. Inconsistent PROMs improvement among patients undergoing ROSA-assisted TKA compared to conventional TKA may be due to confounding factors such as various alignment strategies and implant designs used, and therefore, we cannot definitively conclude that the ROSA system resulted in improved functional outcomes within 1 year of follow-up. Kenanidis et al. [9] was the only study that compared neutral mechanical alignment using both ROSA-assisted and conventional TKA using the same posterior-stabilized implant, while Khan et al. [22] did not state the alignment target for either ROSA-assisted or conventional TKA with various polyethylene insert designs. More uniform studies are required to investigate these different factors' impact on patient-reported outcomes after ROSA-assisted TKA.

There are important limitations to the review that should be acknowledged. First, there was variability between the studies about the type of outcome measures used, alignment strategies, the follow-up period, the patient population and cohorts evaluated, and the analyses performed. Only one study utilized the image-based feature of ROSA. This heterogeneity limits a more robust meta-analysis of the results being conducted. Also, the generalizability of these results may be limited as many of the accuracy and precision studies excluded patients with coronal plane deformities or previous surgery and should be studied in the future. Different validation methods were used by the studies to analyze precision, and accuracy of the robotic platform, with most studies comparing intraoperative plan to intraoperative validation of the device instead of using an external validation tool, which makes this method highly prone to error. Finally, the rotation of the implants was not assessed in any of the included studies, which is known to impact outcomes after TKA. Future studies utilizing cross-sectional imaging are required to evaluate these factors. The main strength of this study was the assessment of a singular robotic-arm assisted system for TKA. To our knowledge, no systematic reviews specifically evaluate and compare the accuracy, precision, and clinical outcomes of the ROSA robotic platform for TKA.

## Conclusions

This is the first systematic review to evaluate the accuracy and precision of the ROSA Robotic System for TKA, which appears to be both highly accurate and precise. This system reduced the number of outliers when compared to conventional TKA instrumentation and resulted in improved clinical outcomes within 1 year of surgery. Future studies with a more robust methodology and studies investigating the impact of polyethene insert design, alignment strategy, and rotational assessments are needed to help understand the clinical benefits of this semi-active collaborative platform.

## Funding

This study was funded by 10.13039/100012630Zimmer Biomet (A+8742, Auckland City Hospital).

## Conflicts of interest

A. P. Monk is a paid consultant for Zimmer Biomet, Smith and Nephew, and FormusLabs; receives research support from Zimmer Biomet; and is a board/committee member of ISAKOS arthroplasty. All other authors declare no potential conflicts of interest.

For full disclosure statements refer to https://doi.org/10.1016/j.artd.2024.101451.

## CRediT authorship contribution statement

**Faseeh Zaidi:** Writing – review & editing, Writing – original draft, Visualization, Validation, Methodology, Investigation, Formal analysis, Data curation, Conceptualization. **Craig M. Goplen:** Writing – review & editing, Methodology, Investigation, Formal analysis, Data curation, Conceptualization. **Scott M. Bolam:** Writing – review & editing, Formal analysis. **Andrew P. Monk:** Writing – review & editing, Supervision, Conceptualization.

## Figures and Tables

**Appendix B:** Summary of quality assessment of included studies.

Author	Year	Q1	Q2	Q3	Q4	Q5	Q6	Q7	Q8	Q9	Q10	Q11	Q12	Q13	Q14	Score	Quality rating
Batailler et al. [10]	2023	Yes	Yes	Yes	Yes	No	Yes	Yes	Other	Yes	No	Yes	No	Other	No	8	Fair
Bolam et al. [17]	2022	Yes	Yes	Yes	Yes	No	Yes	Yes	Other	Yes	Other	Yes	No	Other	No	8	Fair
Charette et al. [18]	2022	Yes	Yes	Other	No	No	Yes	Yes	Other	Yes	Other	Yes	No	Yes	No	7	Fair
Haffar et al. [19]	2021	Yes	Yes	Other	Yes	Yes	Yes	Yes	Other	Yes	Other	Yes	No	No	Yes	9	Fair
Hasegawa et al. [20]	2022	Yes	Yes	Other	Other	Yes	Yes	Yes	Other	Yes	Other	Yes	No	Other	No	7	Fair
Kenanidis et al. [9]	2023	Yes	Yes	Other	Yes	No	Yes	Yes	Other	Yes	Other	Yes	No	Yes	No	8	Fair
Kenanidis et al. [21]	2023	Yes	Yes	Yes	No	No	Yes	Yes	Other	Yes	Other	Yes	No	Yes	No	8	Fair
Khan et al. [22]	2023	Yes	Yes	No	No	No	Yes	Yes	Other	Yes	Other	Yes	No	No	No	6	Fair
Lonner et al. [23]	2022	Yes	No	Other	Other	No	Yes	Yes	Other	Yes	Other	Yes	No	Other	No	5	Fair
Mancino et al. [6]	2023	Yes	Yes	Yes	Yes	Yes	Yes	Yes	Other	Yes	Other	No	No	No	Yes	9	Fair
Parratte et al. [8]	2023	Yes	Yes	Other	Yes	Yes	Yes	Yes	Other	Yes	Other	Yes	No	Yes	No	9	Fair
Parratte et al. [24]	2019	Yes	Yes	Other	Other	No	Yes	Yes	Other	Yes	Other	Yes	No	Yes	No	7	Fair
Rossi et al. [12]	2023	Yes	Yes	Yes	Yes	No	Yes	Yes	Other	Yes	Other	Yes	No	Yes	No	9	Fair
Schrednitzki et al. [11]	2023	Yes	Yes	No	Yes	No	Yes	Yes	Other	Yes	Other	Yes	No	Yes	No	8	Fair
Seidenstein et al. [25]	2021	Yes	Yes	Other	Other	Yes	Yes	Yes	Other	Yes	Other	Yes	No	Other	No	7	Fair
Shin et al. [26]	2022	Yes	Yes	Other	Yes	Yes	Yes	Yes	Other	Yes	Other	Yes	No	Yes	No	9	Fair
Vanlommel et al. [27]	2021	Yes	Yes	Yes	Yes	No	Yes	Yes	Other	Yes	Other	Yes	No	No	No	8	Fair

## Appendix A: Database search strategy

### PubMed

Date of search: 20 June 2023

Search strategy 243 articles yielded

1. “robot” [All fields]
2. “robotic” [All fields]
3. “robotic surgical procedure” [MeSH terms]
4. “robotic arm assisted” [All fields]
5. 1 OR 2 OR 3 OR 4
6. “arthroplasty, replacement, total knee” [MeSH terms]
7. “arthroplasty” [MeSH terms]
8. 6 OR 7
9. 5 AND 8

### OVID MEDLINE: 1946 – December 2022

Date of search: 20 June 2023

Search strategy 266 articles yielded

1. Osteoarthritis, Knee/
2. (Osteoarthritis Knee or OA knee or Knee arthrosis patient or osteoarthritic knee).tw,kf.
3. 1 or 2
4. Robotic Surgical Procedures/
5. Robotics/
6. (Robo or Robot∗ or RATKA or RATKR or Robotic arm∗ or assisted Robotic device∗ or Robotic surgical device∗ or Robotic-aided surger∗).tw,kf.
7. 4 or 5 or 6
8. Arthroplasty, Replacement, Knee/
9. ([conventional or traditional or primary or total] adj3 [knee replacement or knee arthroplasty or knee surgery or knee resur-fac∗]).tw,kf.
10. 8 or 9
11. 3 and 7 and 10
12. Limit 11 to English language

### EMBASE

Date of search: 20 June 2023

Search strategy 182 articles yielded

1. exp knee osteoarthritis/
2. (Osteoarthritis knee or OA knee or knee arthrosis patient or osteoarthritic knee).tw,kf.
3. 1 or 2
4. Robot assisted surgery/
5. exp robotics/
6. exp robot/
7. (Robo or Robot∗ or Robotic or Robotic surgery or RATKA or RATKR or Robotic arm∗ or assisted Robotic device∗ or Robotic surgical device∗ or Robotic-aided surger∗).tw,kf.
8. 4 or 5 or 6 or 7
9. Total knee arthroplasty/ or total knee prosthesis/
10. exp knee replacement/
11. ([conventional or traditional or total or primary] adj3 [knee replacement or knee arthroplasty or knee surgery or knee resur-fac∗]).tw,kf.
12. 9 or 10 or 11
13. 3 and 8 and 12
14. Limit 13 to English language

### Cochrane Library

Date of search: 20 June 2023

Search strategy 96 articles yielded

1. [mh “knee osteoarthritis”]
2. (Osteoarthritis knee or OA knee or Knee arthrosis patient or osteoarthritic knee):ti,ab,kw
3. #1 OR #2
4. [mh ˆ“robot assisted surgery”]
5. [mh “robotics”]
6. [mh “robot”]
7. (Robo or Robot∗ or Robotic or Robotic surgery or RATKA or RATKR or Robotic arm∗ or assisted Robotic device∗ or Robotic surgical device∗ or Robotic-aided surger∗):ti,ab,kw
8. #4 or #5 or #6 or #7
9. [mh “Arthroplasty”]
10. ([conventional or traditional or primary or total] NEAR/3 [knee replacement or knee arthroplasty or knee replacement or knee arthroplasty or knee surgery or knee resurfac∗]):ti,ab,kw
11. #9 OR #10
12. #3 AND #8 AND #11

## Appendix A Supplementary Data

Conflict of Interest Statement for Monk

Conflict of Interest Statement for Goplen

Conflict of Interest Statement for Zaidi

Conflict of Interest Statement for Bolam

PRISMA 2020 Checklist
