# Supplementary material for: Accuracy and Outcomes of a Novel Cut-Block Positioning Robotic-Arm Assisted System for Total Knee Arthroplasty: A Systematic Review and Meta-Analysis
Source: Arthroplast Today. 2024 Aug 4;29:101451. doi: 10.1016/j.artd.2024.101451 (PMC11345934; doi:10.1016/j.artd.2024.101451)
Supplement: Conflict of Interest Statement for Zaidi [file mmc3.docx]

# INDIVIDUAL CONFLICT OF INTEREST STATEMENT

***American Association of Hip and Knee Surgeons***

(Adopted from the American Academy of Orthopaedic Surgeons disclosure statement)

The following form **must be filled out completely and submitted by each author (example, 6 authors, 6 forms).**

**All items require a response. If there is no relevant disclosure for a given item, enter "*None*.”**

**Manuscript Title** Accuracy and Outcomes of a Novel Cut-block Positioning Robotic‐Arm Assisted System for Total Knee Arthroplasty: A Systematic Review and Meta-Analysis

1. Royalties from a company or supplier (The following conflicts were disclosed)

*None*

2. Speakers bureau/paid presentations for a company or supplier (The following conflicts were disclosed)

*None*

3A. Paid employee for a company or supplier (The following conflicts were disclosed)

*None*

3B. Paid consultant for a company or supplier (The following conflicts were disclosed)

*None*

3C. Unpaid consultants for a company or supplier (The following conflicts were disclosed)

*None*

4. Stock or stock options in a company or supplier (The following conflicts were disclosed)

*None*

5. Research support from a company or supplier as a Principal Investigator (The following conflicts were disclosed)

*None*

6. Other financial or material support from a company or supplier (The following conflicts were disclosed)

*None*

7. Royalties, financial or material support from publishers (The following conflicts were disclosed)

*None*

8. Medical/Orthopaedic publications editorial/governing board (The following conflicts were disclosed)

*None*

9. Board member/committee appointments for a society (The following conflicts were disclosed)

*None*

**Each author must sign AND print or type his/her name, date and submit a separate form**

In addition, one BLINDED Conflict of Interest form (no author names used) should be submitted per manuscript with all author disclosures.


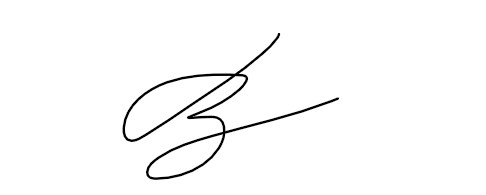


Faseeh Zaidi 29/09/23

Author Name (Print or Type) Author Signature Date
